# Supplementary material for: Numerical and biomass growth study of Bulimulus bonariensis (Rafinesque, 1833) (Gastropoda: Bulimulidae) under laboratory conditions
Source: PeerJ. 2024 Jan 25;12:e16803. doi: 10.7717/peerj.16803 (PMC10822139; doi:10.7717/peerj.16803)
Supplement: Supplemental Information 2 [file peerj-12-16803-s002.pdf]

Secretaría de Investigación y Transferencia
FACULTAD DE CIENCIAS NATURALES Y MUSEO

UNIVERSIDAD NACIONAL DE LA PLATA

La Plata, 16 de junio de 2023

Se deja constancia que el COMITÉ INSTITUCIONAL PARA EL CUIDADO Y USO DE ANIMALES DE ESTUDIO (CICUAE) de la Facultad de Ciencias Naturales y Museo de la Universidad Nacional de La Plata, evaluó el protocolo presentado por la **Dra. Stella Maris Martín**, titulado **Crecimiento numérico y en biomasa de *Bulimulus bonariensis* (Rafinesque, 1833).**

Este comité considera que el mencionado protocolo se ajusta al reglamento del CICUAE y por lo tanto lo considera **APROBADO**.

Número de protocolo: **009.06.2023**.

Dr. Diego Montalti
CICUAE

Dra. Sandra Torrusio
Secretaría de Investigación y Transferencia
Fac. Ciencias Naturales y Museo
UNLP
